# Pathological and immune features of non-tuberculous mycobacteria and *Mycobacterium tuberculosis* cutaneous/mucosa infections of fifty-four biopsies

**DOI:** 10.3389/fcimb.2025.1664902

**Published:** 2025-10-20

**Authors:** Xiao Ying Zhang, Ying Li, Jing Zhao, Fang Qi Deng, Xu Hua Tang, Jin Fu Li, Wen Ting Yang, Han Wen Jiang, Jin Hua He, Zhuo Wang

**Affiliations:** ^1^ Department of Pathology, The Affiliated Panyu Central Hospital of Guangzhou Medical University, Guangzhou, Guangdong, China; ^2^ Department of Pathology, The First Affiliated Hospital of Sun Yat-sen University, Guangzhou, Guangdong, China; ^3^ Shenzhen Clinical Medical College, Guangzhou University of Chinese Medicine, Guangzhou, China; ^4^ Department of Dermatology, The First Affiliated Hospital of Sun Yat-sen University, Guangzhou, Guangdong, China; ^5^ Department of Pathology, Yunkang Group Limited, Guangzhou, Guangdong, China; ^6^ Department of Central Laboratory, The Affiliated Panyu Central Hospital of Guangzhou Medical University, Guangzhou, Guangdong, China

**Keywords:** NTM, MTB, cutaneous, granuloma, macrophages, PD-L1

## Abstract

**Introduction:**

The histopathologic changes associated with cutaneous non-tuberculous mycobacteria (NTM) infections closely resemble those seen in *Mycobacterium tuberculosis* (MTB) infections, often leading to confusion. This study aimed to enhance clinicopathologic diagnosis and clarify the pathogenesis of NTM and MTB infections by comparing the clinicopathologic features and immunohistochemistry.

**Methods:**

We conducted a detailed observation and comparative analysis of histomorphological features in 27 biopsies of cutaneous/mucosa NTM infections and 27 biopsies of MTB infections, focusing on macrophage distribution and the infiltration of various macrophage subpopulations.

**Results:**

Our findings revealed that NTM disease was more prone to developing small vessel hyperplasia, dilation, congestion, and interstitial edema compared to tuberculosis (TB). Additionally, the counts of lymphocytes, plasma cells, and neutrophils were significantly higher in NTM infections than in TB. NTM disease was primarily characterized by non-necrotizing granulomas, whereas TB was mainly associated with caseous necrotizing granulomas. Distinct macrophage subpopulations were observed in different lesion regions. granuloma epithelioid macrophages induced by NTM infections primarily expressed CD68 and CD206, while macrophages in non-granulomatous regions predominantly expressed CD163. This suggests that these macrophages belong to different subpopulations with distinct roles. Moreover, the positivity rate of PD-L1 in mononuclear inflammatory cells was notably elevated in both NTM and MTB infections.

**Discussion:**

The similarities and differences in histopathological features, macrophage responses, and immune markers between NTMD and TB provide valuable insights into their pathogenic mechanisms. Understanding these variations could inform better diagnostic and therapeutic strategies for managing NTM infections.

## 1 Introduction

The term non-tuberculous mycobacteria (NTM) refers to a diverse group of mycobacteria distinct from the “*Mycobacterium tuberculosis* complex” and “*Mycobacterium leprae*.” Previously, this group included terms like “atypical mycobacteria” and “atypical acid-resistant mycobacteria.” To date, more than –266 NTM species and 14 subspecies have been identified, most of which are commensal bacteria, with only a few being pathogenic to humans under certain conditions (Smith et al., 2016; Yoon et al., 2017; Santin et al., 2018; Furuuchi et al., 2019; Winthrop et al., 2020; Yazdanmanesh et al., 2024). Tuberculosis (TB), caused by *Mycobacterium tuberculosis* (MTB), exhibits similar histopathological changes to those seen in NTM infections, leading to potential diagnostic confusion. However, significant differences exist in clinical treatment and prognosis, making accurate pathological diagnosis crucial for guiding clinical management.

Macrophages can be broadly classified into two categories: M1 and M2, based on their inflammatory roles. Classically activated macrophages (M1) are induced by lipopolysaccharide (LPS) and interferon-γ (IFN-γ) (Dolezalova et al., 2022), playing a critical role in anti-infective responses during the early stages of the disease. Conversely, alternatively activated macrophages (M2), stimulated by IL-4 or IL-13, are significant in chronic disease progression and fibrosis. NTM and MTB can exist not only intracellularly but also extracellularly, and they can survive phagocytosis, and macrophages are essential in inhibiting and killing these pathogens. However, the distinct roles of M1 and M2 macrophages in this context remain unclear.

To investigate these differences, we conducted comprehensive histomorphological examinations of 27 biopsies of cutaneous/mucosa NTMD and 27 biopsies of TB. While both NTMD and TB were primarily present as granulomatous lesions and non-specific chronic suppurative inflammation, distinct pathomorphological differences were identified in the epidermis, dermis, and subcutaneous adipose tissue. Our analysis focused on macrophage infiltration and differences in PD-L1 (programmed death ligand 1) expression in immune cells.

## 2 Materials and methods

### 2.1 Case selection and clinicopathologic evaluation

We analyzed 27 biopsies of cutaneous/mucous NTMD and 27 biopsies of TB diagnosed between December 2, 2013 and May 17, 2023. 26 biopsies of cutaneous NTMD and 26 biopsies of TB were from the First Affiliated Hospital of Sun Yat-sen University, 1 biopsy of cutaneous NTMD and 1 biopsy of TB were from the Affiliated Panyu Central Hospital of Guangzhou Medical University. The inclusion criteria comprised cases of cutaneous/mucous NTMD and TB. The diagnosis was based on clinical presentation, imaging, pathogenesis, pathological examination, metagenomics next-generation sequencing (mNGS), and tuberculosis polymerase chain reaction (TB-PCR) testing (Sardiña et al., 2020; Epperson et al., 2024). Cutaneous/mucous tissue, cutaneous pus, blood, and bronchoalveolar lavage fluid (BALF) were collected for acid-fast bacilli (AFB) culture, mNGS, and TB-PCR testing. mNGS was used as a high-resolution reference method, mNGS plays a critical role in the identification and diagnosis of infectious diseases. However, mNGS still presents several limitations, such as FFPE contamination, detection threshold, and semiquantitative interpretation challenges (Appak et al., 2018; Huang et al., 2019; Zhou et al., 2019; Dulanto Chiang and Dekker, 2020; Qian et al., 2020). In this cohort, all 27 biopsies of NTMD were diagnosed by mNGS, among which 12 biopsies underwent AFB culture, with a positive rate of approximately 58.3% (7/12). All 27 biopsies of tuberculosis (TB) were confirmed by mNGS and TB-PCR, among which 10 biopsies underwent AFB culture, with a positive rate of approximately 60.0% (6/10).

Specimens were fixed in 4% neutral formaldehyde, routinely dehydrated, clarified, paraffin-embedded, and cut into 3 μm thick sections. These sections were stained with hematoxylin and eosin (HE) and subjected to acid-fast staining. Two dermatopathologists with subspecialty expertise conducted independent morphological analyses to assess differences between the groups.

### 2.2 Immunohistochemistry

Immunohistochemical staining was performed on 4 μm sections cut from formalin-fixed, paraffin-embedded tissue using an automated immunostainer. The primary monoclonal antibodies used included CD68 (1:100, mouse monoclonal, clone KP1, catalog No. ZM-0060; OriGene), CD86 (1:100, rabbit monoclonal, clone E2G8P; Cell Signaling), CD163 (RTU, mouse monoclonal, clone 10D6, catalog No. ZM-0428; OriGene), CD206 (1:1000, rabbit monoclonal, catalog No. 18704-1-AP; Proteintech), and PD-L1 (1:50, mouse monoclonal, clone 22C3, catalog No. M3653; Dako). The cells were counted manually by two individuals (X-Y Z and Z W). In cases of discrepancies between the two evaluators, the slides were re-examined and recounted to reach a consensus.

The percentage of positive macrophages was calculated for CD68, CD86, CD163, and CD206, based on the total number of macrophages. PD-L1 expression was assessed by counting PD-L1 stained cells (lymphocytes and macrophages/histiocytes) relative to the total number of live mononuclear inflammatory cells, expressed as a percentage. Membrane staining intensity was considered PD-L1 expression, including in multinucleated giant cells. In PD-L1 assessment, there is currently no standardized criteria for inflammatory lesions. Therefore, we adopted the ≥10% cut-off in immune cells based on the study by Selma Emre (Emre et al., 2023). Samples were categorized into low PD-L1(+) (less than 10% positivity) and high PD-L1(+) (10% or greater positivity) groups.

### 2.3 Metagenomic next-generation sequencing and analysis

When selecting body fluids or fresh tissue from the infection site for mNGS testing, strict aseptic techniques must be followed during the collection of sterile body fluid/tissue samples to avoid false positives caused by environmental mycobacterial contamination. Samples were aseptically sealed and stored at −20 °C or transported on dry ice to the molecular laboratory for mNGS detection. The samples underwent grinding and cell wall disruption procedures followed by host DNA depletion. Upon completion of pre-processing, nucleic acid extraction was performed using magnetic bead-based methods. RNA and DNA libraries constructed from patient-derived samples were sequenced on an Illumina HiSeq platform in rapid-run mode, achieving a depth of 5 to 10 million single-end reads, each 140 base pairs in length. The raw sequencing data were quality-controlled using fastp (v0.23.2), which included adapter trimming, low-quality read filtering, low-complexity sequence removal, and elimination of excessively short reads. A human reference database was constructed based on GRCh38.p13 and the NCBI nt database to identify and remove human-derived sequences through alignment with bowtie2 (v2.3.5.1). A pathogen database was built using the NCBI RefSeq database (v20221231), FDA-ARGOS v1.0, the Genome Taxonomy Database release 206, and the NCBI GenBank database (v20221231). Non-human sequences were aligned to the pathogen database using the Burrows-Wheeler Aligner (BWA, v0.7.17-r1198-dirty). Contaminating sequences were filtered using a laboratory background database, followed by taxonomic classification and statistical analysis (in-house script) to obtain species-specific read counts, relative abundance, and genome coverage. All detected read counts were normalized to 20 M reads. For specific pathogens such as NTM and MTB, the detection of one unique species-specific read was considered positive (Naccache et al., 2014; Schlaberg et al., 2017; Han et al., 2022).

### 2.4 Polymerase chain reaction

Paraffin-embedded tissues were sectioned at 5 μm thickness, and DNA was extracted using the Paraffin-Embedded Tissue Genomic DNA Extraction Kit (Qiagen). The concentration and purity of extracted DNA were assessed by UV spectrophotometry. Subsequently, samples underwent an MTB assay according to the instructions of the Mycobacterium tuberculosis DNA fluorescence diagnostic kit (PCR-fluorescence probing, Sansure Biotech Inc.). Positive and negative controls were established to ensure the accuracy and reliability of results (Choi et al., 2012; Lim et al., 2019; Sawatpanich et al., 2022).

### 2.5 Statistical analyses

Statistical analysis was performed using SPSS 27.0 software. Categorical variables were presented as frequencies and percentages. The Fisher’s exact test was used to compare pathological features, immune cell infiltration, granuloma types, and PD-L1 expression on immune cells. *P*-values were adjusted for multiple testing using the Benjamini-Hochberg (BH) method, with a significance threshold set at *P* < 0.05. The correlations among the positive rates of macrophage markers (CD68, CD86, CD163, and CD206) were analyzed as follows. The Shapiro-Wilk test (prioritized for sample sizes n ≤ 50) was used to assess normality. Variables with *p* > 0.05 were considered normally distributed, and Pearson correlation coefficients were calculated. For variables failing to meet the normality criterion (*p* ≤ 0.05), nonparametric Spearman correlation was employed. Correlation strength was quantified using *R*-values ranging from –1 to 1, where values closer to ±1 denote a stronger linear association between variables. A *p*-value less than 0.05 was considered statistically significant.

## 3 Results

### 3.1 Clinical findings

A total of 27 biopsies of NTMD were analyzed, with cutaneous/mucosal biopsy specimens obtained from each case (**Table 1**). The biopsies included 8 of *M. marinum*, 6 of *M. abscessus*, 5 of the *M. avium-intracellulare* complex (MAC), 3 of *M. haemophilum*, 3 of *M. avium*, 1 of *M. colombiense*, and 1 of *M. mantenii*. Specimens were collected from various sites: 11 from the limbs, 4 from the face, 2 from the back, 2 from the abdomen, 1 from the neck, 5 from unspecified cutaneous locations, and 2 from the nasal mucosa.

**Table 1:** Clinical findings of NTMD.

No. of biopsies	NTM subtype	Location	History of seafood exposure	Chronic diseases/tumor	Clinical diagnosis	Immunosuppressive agent
1	*M.marinum*	Limb	Yes	None	CNTMD	None
2	*M.marinum*	Limb	Yes	None	CNTMD	None
3	*M.marinum*	Limb	No	Diabetes	CNTMD	None
4	*M.marinum*	Limb	No	None	CNTMD	None
5	*M.marinum*	UCL	No	None	CNTMD	None
6	*M.marinum*	UCL	No	None	CNTMD	None
7^a^	*M.marinum*	Limb	No	None	CNTMD	None
8^a^	*M.marinum*	Limb	No	None	CNTMD	None
9	*M. abscessus*	Face	No	None	CNTMD	IARAIS
10	*M. abscessus*	Limb	No	None	CNTMD	None
11	*M. abscessus*	Back	No	None	DNTMD	IARAIS
12^b^	*M. abscessus*	Face	No	None	DNTMD	IARAIS
13^b^	*M. abscessus*	Abdomen	No	None	DNTMD	IARAIS
14^b^	*M. abscessus*	Neck	No	None	DNTMD	IARAIS
15	MAC	Nasal mucosa	No	DILI	DNTMD	None
16^c^	MAC	Limb	No	None	DNTMD	IARAIS
17^c^	MAC	UCL	No	None	DNTMD	IARAIS
18^c^	MAC	UCL	No	None	DNTMD	IARAIS
19^d^	MAC	UCL	No	None	CNTMD	IARAIS
20	*M.haemophilum*	Limb	Yes	None	CNTMD	None
21^e^	*M.haemophilum*	Back	No	Lymphoma	DNTMD	None
22^e^	*M.haemophilum*	Abdomen	No	Lymphoma	DNTMD	None
23	*M. avium*	Nasal mucosa	No	None	CNTMD	None
24	*M. avium*	Limb	No	None	CNTMD	None
25	*M. avium*	Face	No	None	DNTMD	IARAIS
26	*M. colombiense*	Limb	No	Castleman Diseases	CNTMD	None
27^d^	*M.mantenii*	Face	No	None	CNTMD	IARAIS

Biopsies (7^a^, 8^a^), (12^b^, 13^b^, 14^b^), (16^c^, 17^c^, 18^c^), (21^e^, 22^e^) represent different biopsy sites from the same patient.

Biopsies 19^d^ and27^d^ represent different periods of cutaneous biopsies from the same patient, who first had an infection with MAC, and 2 years later had an infection with *M. mantenii*.

F, Female; M, Male; MAC, *M. avium-intracellulare* complex; UCL, Unspecified Cutaneous Location; DILI, Drug-Induced Liver Injury; CNTMD, Cutaneous NTMD; DNTMD, Disseminated NTMD; IARAIS, IFN-γ autoantibody-related adult immunodeficiency syndrome.

The 27 biopsies represented 20 patients with an average age range of 41–72 years (mean age 57.9, median age 59). Most patients were middle-aged or elderly, with a male-to-female ratio of 1:1. Five patients had multiple biopsies from different sites either concurrently or at different times. One patient had successive infections with two NTM species: initially with *M. avium-intracellulare* complex in 2020, which was clinically resolved, followed by *M. mantenii* in 2022.

Among the 20 patients, 6 had disseminated NTMD, all of whom had compromised or deficient immune function, including 4 with adult anti-IFN-γ autoantibody-related immunodeficiency syndrome, one with a history of lymphoma, and one with drug-induced liver damage. Among the 14 biopsies of NTM cutaneous infections, 2 had adult anti-IFN-γ autoantibody-associated immunodeficiency syndrome, and one had diabetes. All 6 patients with anti-IFN-γ syndrome received prednisone for anti-inflammatory treatment, and pathological biopsies were performed prior to the initiation of therapy. The NTMD lesions were primarily painless papules, nodules, or plaques, some merging into larger lesions that exhibited bleeding, crusting, and scarring in chronic cases. A few patients had co-infections with human herpesvirus type 4, human herpesvirus type 5, fine cyclic virus, *Pseudomonas aeruginosa*, and *Candida albicans*.

For tuberculosis (TB), 27 cutaneous/mucosal biopsy specimens were obtained from 24 patients, with ages ranging from 8 to 83 years (mean age 40.8, median age 36). This group displayed a wide age distribution, from children to the elderly, also with a male-to-female ratio of 1:1. Three patients underwent cutaneous biopsies from different sites simultaneously. The biopsies from patients with cutaneous manifestations of tuberculosis were collected from the limbs (12), neck (4), chest (2), buttocks (2) and face (1), as well as from other sites including facial cutaneous, the vocal cords, the floor of the mouth, nasal mucosa, and the inner canthus of the eye. The TB cutaneous lesions typically appeared as plaques or nodules, dark red in color, and were often accompanied by desquamation, vesiculobullous eruptions, oozing, ulceration, and crusting.

### 3.2 Pathological morphological analysis of NTMD and TB

**Figure 1:**
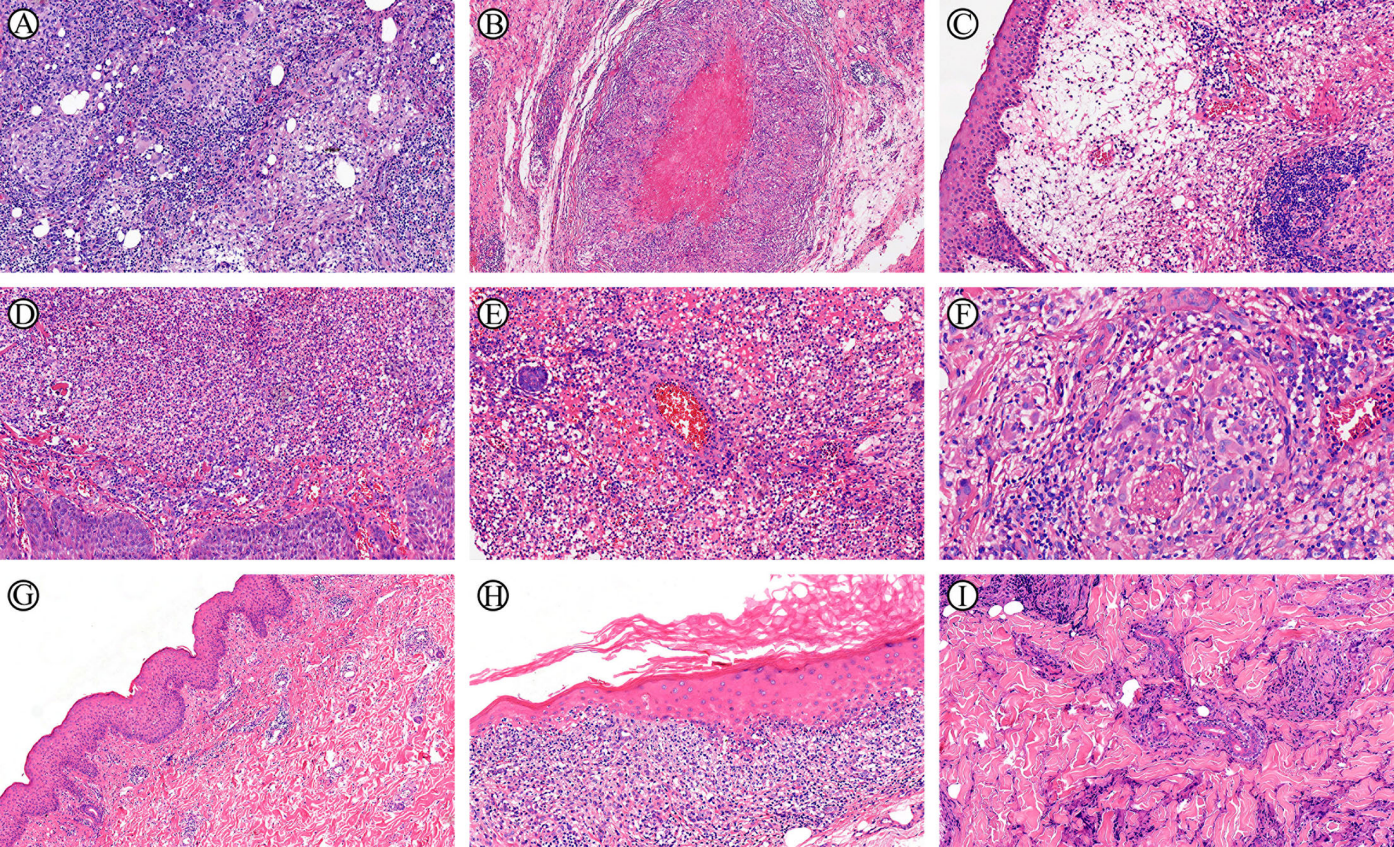
NTMD pathomorphological characterization of diverse bacterial strains (H&E staining). **(A)** *M. marinum*, organized granulomas (300-500 μm). **(B)** *M. marinum*, granulomas with central fibrinous necrosis. **(C)** *M. marinum*, interstitial edema. **(D)** *M. abscessus*, aggregation of neutrophils and microabscess. **(E)** *M. abscessus*, intravascular neutrophilic infiltration. **(F)** *M. abscessus*, histiocytes containing faintly basophilic granules and filamentous cellular debris. **(G)** MAC, sparse inflammatory cell infiltration. **(H)** *M.haemophilum*, interface dermatitis. **(I)** *M. avium*, prominent, coarse collagen proliferation.

**Figure 2:**
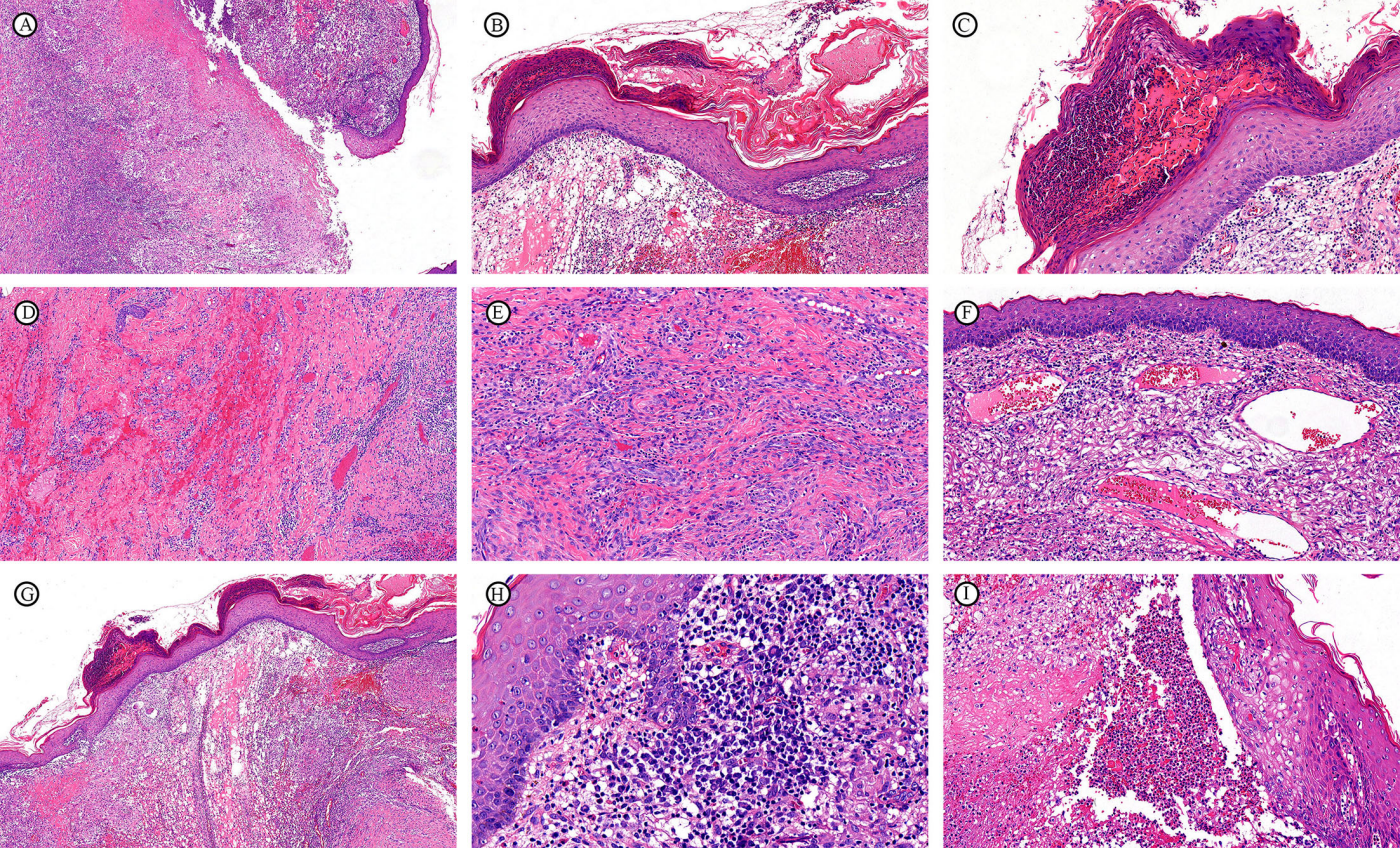
Pathological morphological features of TB (H&E staining). **(A)** Cutaneous ulcers. **(B)** Hyperkeratosis and parakeratosis of the epidermis with serous exudate. **(C)** Munro microabscesses. **(D)** Interstitial hemorrhage. **(E)** Proliferation of microvessels. **(F)** Dilation and congestion of microvessels. **(G)** Moderate interstitial edema. **(H)** Patchy infiltration of plasma cells. **(I)** Microabscess.

A comparative analysis of the pathomorphological characteristics of the epidermis, dermis, and subcutaneous adipose tissue was conducted (Supplemental Digital Content **Supplementary Figure S1**, which demonstrates gross pathological morphological features of NTMD and TB), focusing on subtle features (**Figures 1**, **2**). Epidermal characteristics included hyperplasia, ulceration, parakeratosis/hyperkeratosis, granulocytic exudates, plasma exudates, and spongy edema. Dermal and subcutaneous features included interstitial edema, hemorrhage, microvessels hyperplasia, microvessels dilation and congestion, vasculitis, necrosis, and destruction of appendages. Four NTMD biopsies and eight TB biopsies exhibited loss of the epidermis due to ulceration or crusting, while two NTMD biopsies and two TB biopsies from nasal mucosa. Consequently, fewer biopsies were available for evaluation of epidermal-related features. A comparison of the features revealed that NTMD exhibited more microvessels hyperplasia, dilation, congestion, and interstitial edema than TB, although these differences did not reach statistical significance (**Table 2**). Pseudoepitheliomatous hyperplasia was observed in NTMD (3/27, 11.1%) and TB (2/27, 7.4%), with no significant difference. Epidermal hyperplasia and ulceration were present in both NTMD and TB biopsies, as were parakeratosis/hyperkeratosis, granulocytic exudation, plasma exudation, spongiotic edema, hemorrhage, vasculitis, and destruction of appendages, with no significant differences noted.

**Table 2:** Comparison of pathological features between NTMD and TB.

Pathological feature	NTMD number of positive case/number of biopsies tested (percentage positive)	TB number of positive case/number of biopsies tested (percentage positive)	Adj. *p*
Epidermal hyperplasia	16/21(76.2%)	10/17(58.8%)	0.738
Ulceration	6/21(28.6%)	6/17(35.3%)	0.897
Hyperkeratosis/Parakeratosis	14/21(66.7%)	9/17(52.9%)	0.873
Granulocytic exudates	10/23(43.5%)	6/17(35.3%)	0.897
Plasma exudates	3/23(13.0%)	3/17(17.7%)	1.000
Spongy edema	5/23(21.7%)	1/17(5.9%)	0.647
Appendage destruction	19/27(70.4%)	12/17(70.6%)	1.000
Hemorrhage	14/27(51.9%)	11/27(40.7%)	0.879
Microvessels hyperplasia	20/27(74.1%)	12/27(44.4%)	0.309
Microvessels dilation and congestion	18/27(66.7%)	9/27(33.3%)	0.309
Interstitial edema	19/27(70.4%)	13/27(48.2%)	0.647
Vasculitis	0/27(0%)	2/27(7.4%)	0.873

### 3.3 NTMD pathomorphological characterization of diverse bacterial strains

A comparison of the pathomorphological features of NTMD from seven different strains revealed distinct findings (**Figure 1**, Supplemental Digital Content **Supplementary Table S2**, which demonstrates NTMD pathomorphological characterization of diverse bacterial strains): (i) *M. marinum* exhibited two distinct histopathological patterns: The first was characterized by well-formed granulomas (300 -500 μm) composed of abundant epithelioid cells, often with central fibrinous necrosis. The second pattern featured marked interstitial edema, hemorrhage, and loosely distributed epithelioid cells without distinct nodular formation, accompanied by peripheral granulation tissue proliferation. (ii) *M. abscessus* infection consistently demonstrated neutrophilic infiltration. In severe cases, aggregates of neutrophils within newly formed blood vessels were observed. A distinctive feature was the presence of foamy histiocytes with abundant pale cytoplasm, these foamy histiocytes exhibit the distinctive feature of containing faintly basophilic granules and filamentous cellular debris within their cytoplasm. (iii) MAC infection typically demonstrate relatively sparse inflammatory cell infiltration. Histopathological examination reveals only mild neutrophilic infiltration. (iv) A distinctive histopathological feature of *M.haemophilum* infection is the presence of interface dermatitis. (v) *M. avium* infection is characterized by prominent, coarse collagen proliferation, resulting in characteristic keloid-like fibrosis. (vi) *M. colombiense* demonstrated focal interstitial edema, loosely arranged epithelioid cells, hemorrhage, granulation tissue proliferation, and granular necrosis. (vii) *M.mantenii* presented with small clusters of epithelioid cells (100 μm), diffuse neutrophilic infiltration without distinct abscess formation, and minimal intravascular neutrophil aggregation.

### 3.4 Immune cell infiltration in NTMD and TB

Both NTMD and TB primarily present with granulomatous lesions and nonspecific chronic suppurative inflammation. Immune cell types present include lymphocytes, plasma cells, eosinophils, neutrophils, histiocytes, and multinucleated giant cells. Immune cell infiltration was graded on a scale of 0 to 3, where grade 0 indicates absence, grade 1 denotes focal or scattered infiltration, grade 2 signifies multifocal aggregates and grade 3 indicates small patchy or diffuse infiltration. The histopathological grading of neutrophilic infiltration is categorized as: grade 1 indicates neutrophils are sparsely distributed with low cellular density, grade 2 indicates moderate neutrophil density with formation of small neutrophil foci, grade 3 indicates dense, confluent neutrophilic infiltration with microabscess or abscess formation. A comparative analysis revealed significant differences in the infiltration of lymphocytes, plasma cells, neutrophils, and histiocytes between NTMD and TB, with no significant differences in eosinophils or multinucleated giant cell distribution (**Table 3**). The number of lymphocytes, plasma cells, and neutrophils was significantly higher in NTMD compared to TB. Neutrophil infiltration was observed in all NTMD biopsies, mostly in small focal distributions (grade 1, 14/27, 51.85%). A few neutrophilic microabscesses (grade 3) were seen in 4 biopsies (4/27, 14.8%), while neutrophils were absent in 29.63% of TB biopsies. Only one case of TB exhibited neutrophilic microabscesses (grade 3). In addition to forming granulomas as epithelioid cells, histiocytes in NTM biopsies could also be seen as foamy histiocytes scattered or patchily distributed in the dermis and subcutaneous tissue (excluding the ulcerated granulation tissue), predominantly in focal distributions (grade 1, 14/27, 51.85%). In TB biopsies, histiocytes were mainly involved in granuloma formation as epithelioid cells, with only a few biopsies (5/27, 18.52%) showing scattered or patchily distributed foamy histiocytes (grade 1) in the stroma (excluding ulcerated granulation tissue).

**Table 3:** Comparison of immune cell infiltration in NTMD and TB.

Immune cell	NTMD (n=27) number of positive case/number of biopsies tested (percentage positive)				TB (n=27) number of positive case/number of biopsies tested (percentage positive)				Adj.*P*
	0 Grade	1 Grade	2 Grade	3 Grade	0 Grade	1 Grade	2 Grade	3 Grade	
Lymphocytes	0(0.0%)	7(25.9%)	14 (51.9%)	6 (22.2%)	0 (0.0%)	20 (74.1%)	7 (25.9%)	0 (0.0%)	0.002
Plasma Cells	1(3.7%)	18(66.7%)	5 (18.5%)	3 (11.1%)	9 (33.3%)	16 (59.3%)	2 (7.4%)	0 (0.0%)	0.013
Eosinophils	17(63.0%)	7(25.9%)	3 (11.1%)	0(0.0%)	15 (55.6%)	12 (44.4%)	0 (0.0%)	0 (0.0%)	0.154
Neutrophils	0(0.0%)	14(51.9%)	9 (33.3%)	4 (14.8%)	8 (29.6%)	15 (55.6%)	3 (11.1%)	1 (3.7%)	0.008
Histiocytes	0(0.0%)	14(51.9%)	8(29.6%)	5(18.5%)	0(0.0%)	5(18.5%)	20(74.1%)	2(7.4%)	0.009
MGC	9(33.3%)	12(44.4%)	5 (18.5%)	1 (3.7%)	3 (11.1%)	19 (70.3%)	3 (11.1%)	2 (7.4%)	0.154

MGC, Multinucleated Giant Cells.

### 3.5 Granuloma formation in NTMD and TB

**Figure 3:**
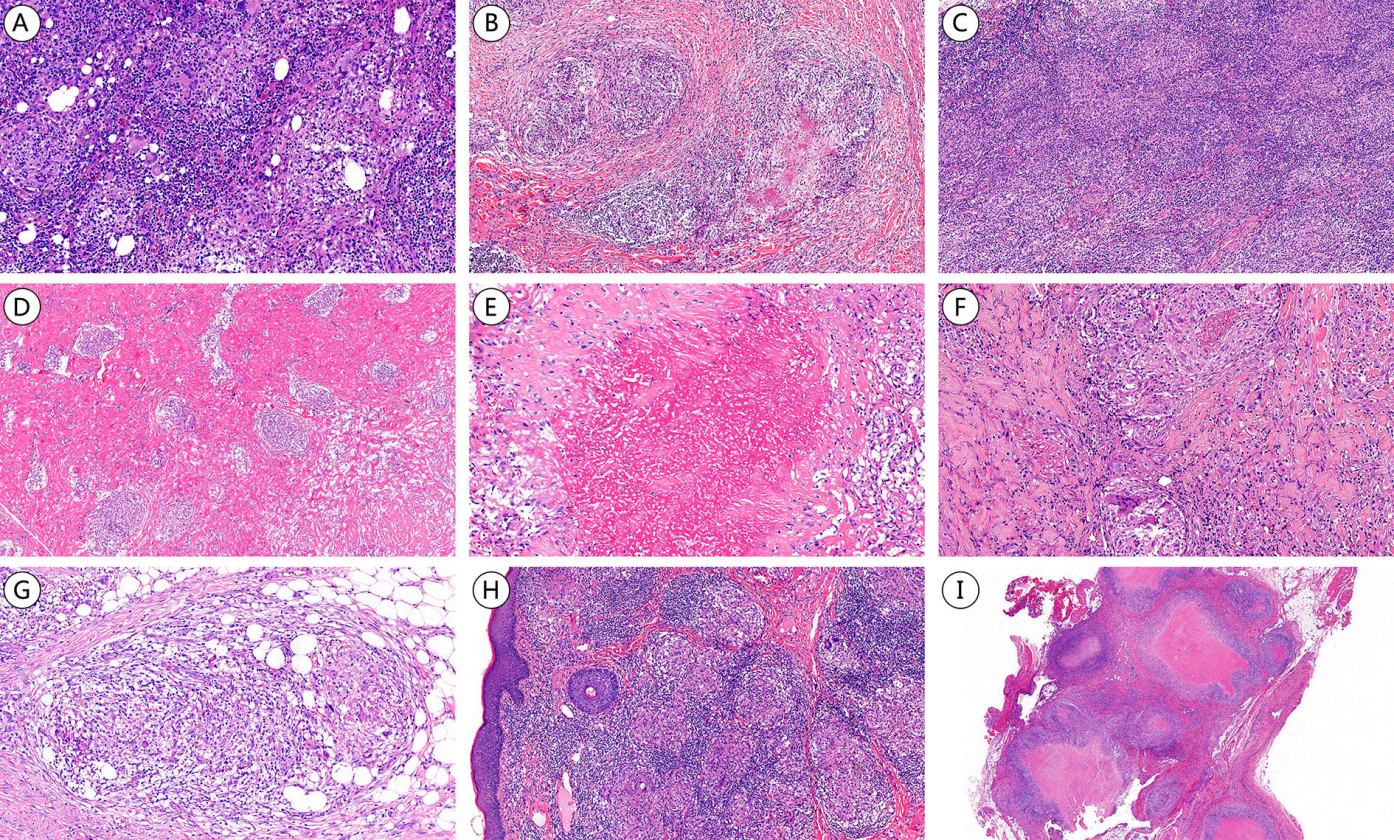
Compares the granuloma morphologies in NTMD and TB (H&E staining). **(A)** *M. marinum*, multiple small non-necrotizing granulomas. **(B)** *M. abscessus*, necrotizing and non-necrotizing granulomas. **(C)** MAC, multiple non-necrotizing granulomas in the nasopharynx (case 15). **(D)** *M. haemophilum*, multiple non-necrotizing granulomas. **(E)** *M. haemophilum*, caseous necrotizing granulomas with thorough necrosis, complete disappearance of cellular outlines and structures, appearing as homogeneous eosinophilic granular material. **(F)** *M. avium*, necrotizing and non-necrotizing granulomas. **(G)** *M. colombiense*, non-necrotizing granulomas. **(H)** *M. tuberculosis*, multiple non-necrotizing granulomas. **(I)** *M. tuberculosis*, caseous necrotizing granulomas.

Granulomatous lesions can be classified as necrotizing or non-necrotizing, based on necrosis presence. Necrotizing granulomas are further categorized into caseous, coagulative, and pyogenic necrosis. NTMD presented with granulomas in 19 of 27 biopsies (70.4%), while TB exhibited granulomas in 26 of 27 biopsies (96.3%), indicating a significantly higher frequency in TB(Supplemental Digital Content **Supplementary Table S1**, which demonstrates comparison of granuloma types between NTMD and TB). Both NTMD and TB can have non-necrotizing or necrotizing granulomas; however, NTMD predominantly exhibited non-necrotizing granulomas (11/27, 40.7%) (**Figures 3A–G**), whereas TB predominantly showed caseous necrotizing granulomas (12/27, 44.4%) (**Figures 3H, I**), with NTMD presenting caseous necrotizing granulomas in 14.8% (4/27). Statistically significant differences were found in both the proportion and classification of granulomas between NTMD and TB.

### 3.6 Analysis of macrophage subsets in NTMD and TB

CD68 is a pan-macrophage marker, M1-type macrophages is marked by the surface marker CD86, while M2-type macrophages are characterized by CD163 and CD206. Macrophages were found distributed in the interstitium, present as scattered histiocytes and granuloma epithelioid macrophages. The mean positivity of macrophages for CD68, CD86, CD163, and CD206 in NTMD was 42.5%, 3.0%, 55.4%, and 43.3%, respectively. In TB, the positivity rates were 41.7%, 2.7%, 44.2%, and 39.0%. Both NTMD and TB exhibited high expression of CD68, CD163, and CD206, with CD163 being the most prominently expressed marker. CD86 was either negative or weakly positive (less than 5% positivity).

**Figure 4:**
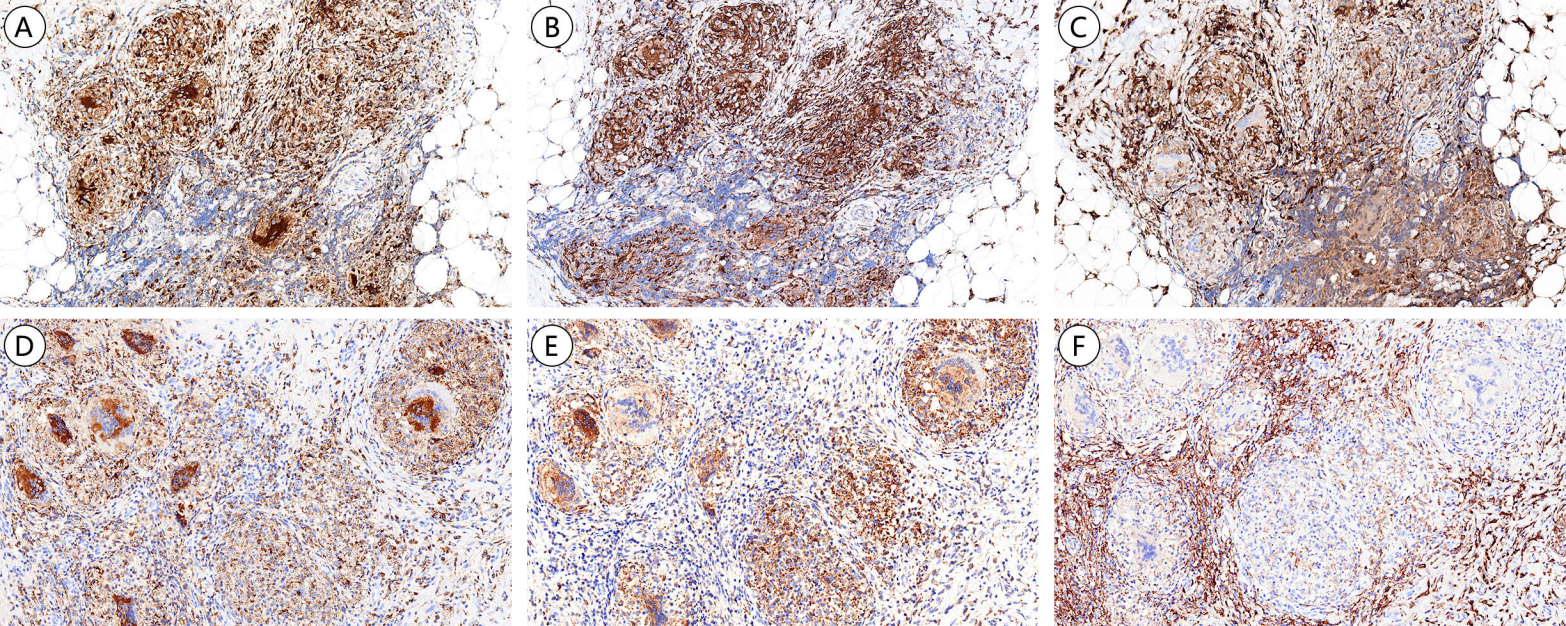
Immunohistochemical expression of different macrophage subsets in NTMD and TB. **(A–C)** *M. avium.* **(A)** CD68 highlights expression primarily in granuloma epithelioid macrophage. **(B)** CD206 expression mainly in granuloma epithelioid macrophage. **(C)** CD163 expression predominantly in interstitial scattered macrophages. **(D–F)** MTB, **(D)** CD68 highlights expression primarily in granuloma epithelioid macrophage. **(E)** CD206 expression mainly in granuloma epithelioid macrophage. **(F)** CD163 expression is mainly in interstitial scattered macrophages.

**Figure 5:**
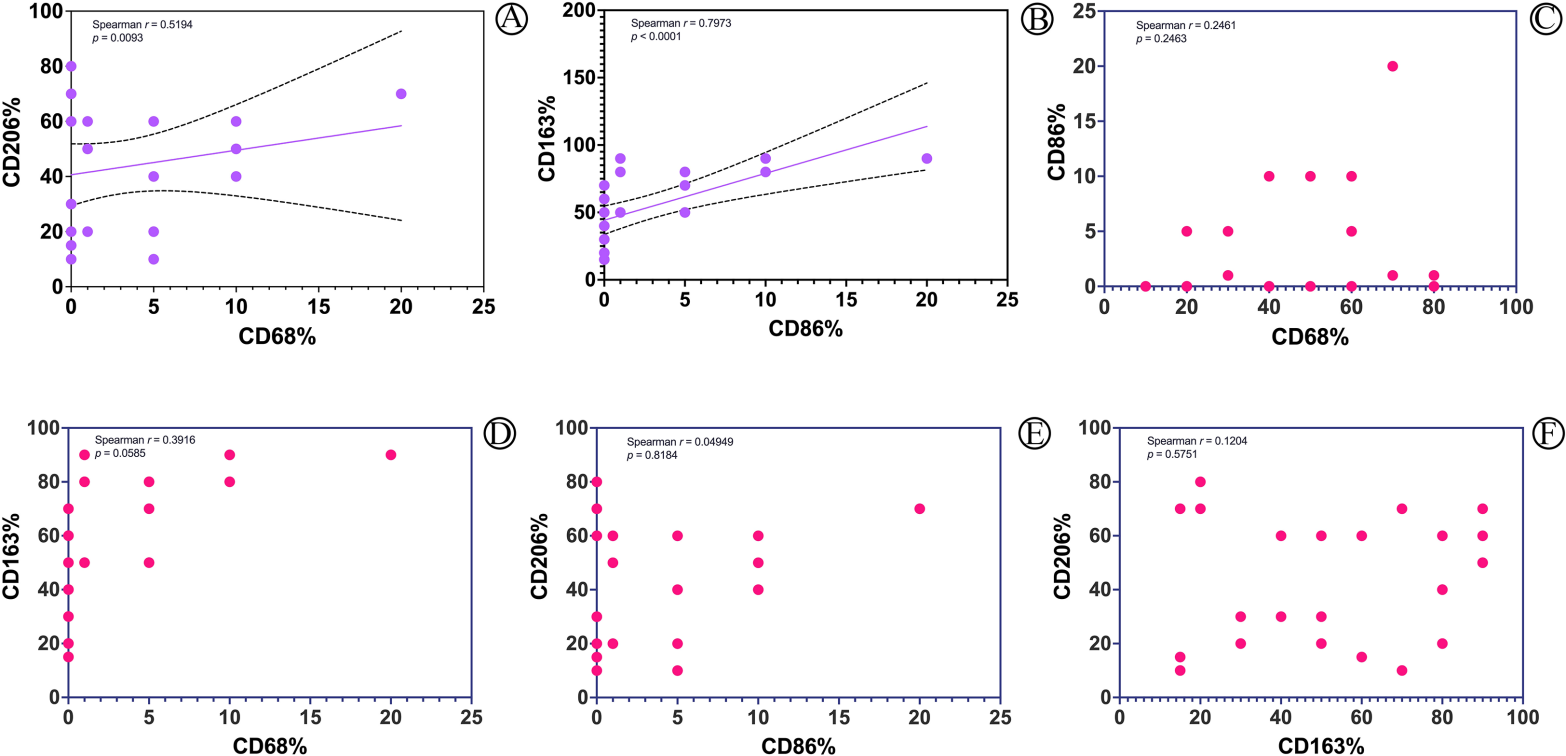
Statistical analysis of the correlation between the positive rates of CD68, CD86, CD163, and CD206 in macrophages in NTMD. **(A)** Significant correlation between CD68 and CD206. **(B)** Significant correlation between CD86 and CD163. **(C–F)** No statistical significance correlations between CD68 and CD86, CD68 and CD163, CD86 and CD206, CD163 and CD206.

In NTMD and TB, CD68 and CD206 were primarily expressed by granuloma epithelioid macrophages, while CD163 was expressed by interstitial scattered macrophages (**Figure 4**). A positive correlation was noted between CD68 and CD206 positivity, as well as between CD86 and CD163 in NTMD, reaching statistical significance (**Figure 5**). No significant correlations were found in TB macrophages (Supplemental Digital Content **Supplementary Figure S2**, which demonstrates statistical analysis of the correlation between the positive rates of CD68, CD86, CD163, and CD206 in macrophages in TB).

### 3.7 Analysis of PD-L1 expression of immune cells in NTMD and TB

**Figure 6:**
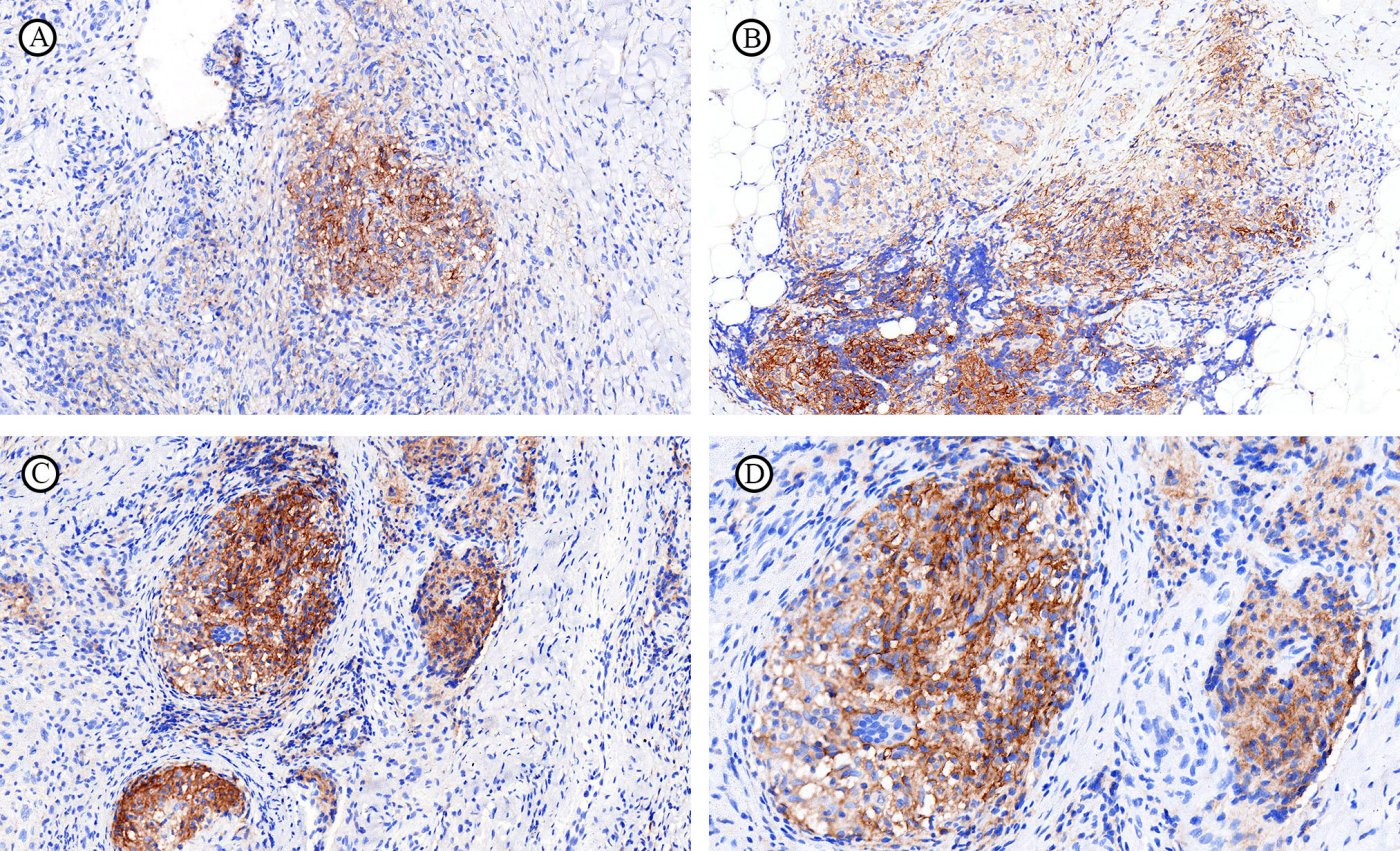
Immunohistochemical PD-L1 expression in NTMD and TB. **(A)** *M. marinum*, granuloma epithelioid macrophage positive for PD-L1. **(B)** *M. avium*, granuloma epithelioid macrophage and mononuclear inflammatory cells positive for PD-L1. **(C, D)** MTB, granuloma epithelioid macrophage positive for PD-L1.

In both NTMD and TB, PD-L1 was expressed by granuloma epithelial cells, lymphocytes, and multinucleated giant cells. Granuloma epithelial cells predominantly expressed PD-L1, whereas lymphocytes and multinucleated giant cells showed low or absent PD-L1 expression (**Figure 6**). In NTMD, mononuclear inflammatory cells exhibited a PD-L1 positivity rate of 0% to 40%, with a mean positivity of 6.4%, which was higher in biopsies of *M. avium* and *M. marinum*. In TB, PD-L1 positivity ranged from 0% to 60% in mononuclear inflammatory cells, with a mean positivity of 14.3%. A higher percentage of mononuclear inflammatory cells in TB (45.8%) demonstrated high PD-L1 expression (≥10%) compared to NTMD (25%), though no significant difference was noted between the two groups (Supplemental Digital Content **Supplementary Table S3**, which demonstrates PD-L1 expression of immune cells in NTMD and TB).

## 4 Discussion

The species identified in 27 biopsies of cutaneous NTMD included *M. marinum*, *M. abscessus*, MAC, *M. haemophilum*, *M. avium*, *M. colombiense*, and *M. mantenii*, with *M. marinum*, *M. abscessus*, and MAC being the most commonly identified Mycobacterium species. Notably, local abscesses of cutaneous and soft tissue are mainly attributed to *M. abscessus*, typically occurring at sites of needle puncture wounds, open injuries, or fractures (Fowler and Mahlen, 2014; Philips et al., 2019), while infections from *M. marinum* and *M. haemophilum* are linked to contact with seafood (Hsueh et al., 2019; Mei et al., 2019). Disseminated NTMD is primarily observed in immunocompromised individuals, particularly those with HIV or other forms of immunodeficiency. Symptoms include persistent or intermittent fever, weight loss, night sweats, and gastrointestinal issues (Nithirungruang et al., 2024; Park et al., 2024). In our cohort, *M. abscessus*, *M. haemophilum*, and MAC were implicated in systemic disseminated NTMD, with 6 of 20 patients being immunocompromised.

The histopathological features of cutaneous NTMD are under-reported in literature (Heid-Picard et al., 2024; Yao et al., 2024). While NTM shares similarities with MTB in bacterial composition and antigens, they are generally less virulent, leading to a milder tissue response and less severe lesions (Nguyen et al., 2024). Main pathological features in NTMD include granulomatous lesions and non-specific chronic suppurative inflammation. The pathological changes generally include: Exudative reactions dominated by lymphocytes, histiocytes, and neutrophils; Proliferative reactions consisting of granulomas formed by macrophages and Langerhans giant cells, sometimes with caseous or coagulative necrosis; Sclerotic reactions involving the regression of inflammatory cells, granuloma atrophy, and collagen fiber proliferation (Nguyen et al., 2024).

NTM species are diverse, and the histopathological changes in various types of infections are not entirely consistent: (i) *M. marinum* infection demonstrated biphasic histologic manifestations, type I (granuloma-predominant) organized epithelioid granulomas with central fibrinoid necrosis, and type II (edema-predominant) a diffuse edematous pattern exhibiting hemorrhagic stroma without discrete granuloma formation. These distinct histopathological patterns likely reflect the host immune response to infection, mirroring the immune spectrum seen in tuberculosis. When host immunity is relatively robust and bacterial burden is low, the predominant pathological manifestation is small, well-formed granulomas. Conversely, in immunocompromised states with high mycobacterial load and enhanced pathogenicity, the pathology shifts toward marked tissue edema with scattered epithelioid cells. Notably, both patterns may coexist within the same lesion, with one predominating and potential interconversion between them. Case 5 exhibited both pathological patterns simultaneously. (ii) *M. abscessus* infection reveals neutrophil-dominated inflammation, Bayer-Garner (Bayer-Garner et al., 2005) suggests that *M. abscessus* exploits neutrophil-rich settings to promote its survival. the foamy histiocytes characterized by their voluminous pale cytoplasm containing distinctive intracytoplasmic inclusions of faintly basophilic granules and filamentous degenerative debris. This unique feature, representing intracellular phagocytosed bacterial and degenerated cellular debris, serves as a diagnostic hallmark for *M. abscessus*. (iii) MAC characteristically shows diminished inflammatory cell infiltration compared to other NTM infections. This observation may suggest that MAC exhibits relatively weaker pathogenicity and elicits a milder host inflammatory response. (iv) *M.haemophilum* infection is characterized by interface dermatitis, a diagnostic morphological feature that demonstrates remarkable specificity when differentiating from other NTM species. Busam (Busam et al., 1999) reveal that infection by *M. haemophilum* can present with nongranulomatous or pauci-granulomatous reactions without necrosis is of note, one biopsy showed an interface dermatitis. (v) *M. avium* infection demonstrates distinctive stromal changes featuring thickened, hyalinized collagen bundles that progress to form pathognomonic keloid-type fibrotic lesions.

Macrophages are the most important cell type in the innate immune response against both NTM and MTB, playing a crucial role throughout the infection process, including establishment, progression, and dissemination. Macrophages can be classified into classically activated M1 type (CAM) and alternatively activated M2 type (AAM) (Lee and Sacks, 2024). M1 macrophages are mainly involved in inflammatory responses and mediating tissue damage, with common surface marker CD86. M2 macrophages can downregulate immune responses, suppress inflammation, and promote tissue repair and remodeling (Zhang et al., 2024), with common surface markers including CD163 and CD206. Additionally, macrophages can regulate their own polarization through autocrine and paracrine signaling, allowing the conversion between M1 and M2 types and utilizing this plasticity to balance the immune microenvironment (Jiang et al., 2024; Luo et al., 2024; Salmaninejad et al., 2024). In our study, both NTMD and TB granulomas highly express CD68 and CD206 (M2 type macrophages), and the scattered macrophages highly express CD163 (M2 type macrophages), indicating involvement in chronic inflammation. The location of CD163 and CD206 expressing macrophages differs: CD163 expressing macrophages are mainly found in non-granuloma regions, while CD206 expressing macrophages are predominantly in the granulomas. There is no significant correlation between their positivity rates. The differing localization of CD163 and CD206 expressing macrophages suggests that these M2 macrophage subsets belong to different subpopulations with distinct functional roles. CD206+ macrophages may be involved in the intracellular growth of the pathogen, while CD163+ macrophages may be involved in immune response modulation. Both NTM and TB macrophages show almost no expression of CD86, with only a few biopsies showing focal weak positivity, possibly due to the lower sensitivity of the CD86 antibody.

Correlation analysis of positivity rates for CD68, CD86, CD163, and CD206 reveals a significant correlation between CD68 and CD206 in NTMD, primarily expressed in granulomas. Although CD86 is expressed at low levels in non-granuloma region macrophages, this study revealed a significant correlation between CD86 and CD163. No statistical significance is found in TB, suggesting that in NTMD, CD68+CD206+ and CD86+CD163+ may represent more independent macrophage subpopulations that warrant further to exploring their potential roles in the pathogenesis of NTMD. NTM and MTB are successful intracellular pathogens that are mainly harbored in macrophages of the host. Although macrophages have several antimicrobial mechanisms to eliminate the intracellular bacteria, NTM and MTB can survive and multiply within them by adjusting and adapting to host immune responses (Mi et al., 2017). PE/PPE family proteins are primarily found in pathogenic mycobacteria species and they are usually expressed during infection and secreted or exposed to the cell surface. All these factors imply that PE/PPE proteins play a critical role in mycobacterial pathogenicity (Fishbein et al., 2015). A crucial factor in MTB’s virulence is the ESX-5 secretion system, which plays an essential role in the transport of PE/PPE proteins, cell wall integrity, and full virulence of MTB (Bottai et al., 2012; Ehtram et al., 2025). The ESX-5 secretion system and PE/PPE proteins collectively facilitate immune evasion and promote intracellular persistence within macrophages.

PD-1 is an inhibitory receptor predominantly expressed on T lymphocytes, which plays a critical role in regulating autoimmunity and self-tolerance. PD-1 is a transmembrane protein expressed on the cell membrane of T cells, B cells, and other immune cells, as well as on tumor cells. PD-L1 immunohistochemistry is a simple and effective method for predicting the efficacy of PD-L1 inhibitors. This technique involves assessing the expression levels of PD-L1 on the surface of tumor cells (TC) or immune cells (IC) to estimate the potential effectiveness of PD-L1 inhibitors. Selma Emre (Emre et al., 2023) investigated the expression of PD-1/PD-L molecules in the lesioned skins of psoriasis patients. the PD-1 and PD-L1 expressions were significantly higher in immune cells than that in the skin samples of the healthy controls. In our experiments, we compared PD-L1 expression in NTMD and TB biopsies by assessing the positivity rate in immune cells. Granuloma epithelioid macrophages, lymphocytes, and multinucleated giant cells can express PD-L1, primarily in granuloma epithelioid macrophages. Non-necrotizing granulomas and necrotizing granulomas both expressed PD-L1, but non-necrotizing granulomas showed stronger expression. 70.8% of NTMD immune cells had a PD-L1 positive rate greater than 1%, with an average positive rate of 6.4%, and higher positive rates were observed in *M. avium* and *M. marinum*. 87.5% of TB immune cells had a PD-L1 positive rate greater than 1%, with an average positive rate of 14.3%. 25% of NTMD immune cells showed high PD-L1 expression (≥10%), and 45.8% of TB immune cells showed high PD-L1 expression. Although there was no significant difference between the two groups, the expression levels still reflected the characteristic that the immune cells’ response in TB is somewhat stronger than in NTMD. NTMD complicated by anti-IFN-γ autoantibody-associated immunodeficiency syndrome immune cells showed low or absent PD-L1 expression. Immunosuppressed patients demonstrated reduced PD-L1 expression on immune cells, although the relationship between PD-L1 expression and immunosuppression remains unclear.

## 5 Conclusion

This study highlights the distinct histopathological features of NTMD compared to TB, NTMD shows higher prevalence of small vessel changes (hyperplasia/dilation/congestion), interstitial edema, and elevated counts of lymphocytes, plasma cells, and neutrophils. The histopathological features of cutaneous NTM infections show distinct patterns: *M. marinum* demonstrates marked interstitial edema, *M. abscessus* exhibits prominent neutrophilic infiltration, *M. avium* infection is characterized by prominent, coarse collagen proliferation, and *M. haemophilum* presents interface dermatitis. NTMD is characterized by non-necrotizing granulomas, while TB features necrotizing granulomas. The granulomas within NTMD predominantly express CD68 and CD206, contrasting with the CD163 expression found in non-granulomatous areas, indicating distinct subpopulations with different roles.

## Data Availability

The datasets presented in this study can be found in online repositories. The names of the repository/repositories and accession number(s) can be found in the article/**Supplementary Material**.

## Supplementary material

Supplementary Figure 1 Gross pathological morphological features of NTMD and TB (H&E staining). **(A–C)** *M. marinum*. **(A)** Pseudoepitheliomatous hyperplasia with significant inflammatory reaction (case 7). **(B)** Significant inflammatory reaction concentrated in the epidermis and superficial dermis (case 8). **(C)** Pseudoepitheliomatous hyperplasia with marked hyperkeratosis (case 1). D-F *M. abscessus*. **(D)** Inflammation was limited to the epidermis and superficial dermis, with relatively mild inflammation (case 13). **(E)** Significant inflammatory reaction with central necrosis and neutrophil aggregation (case 14). **(F)** Inflammation was multifocal and extended deep into the subcutaneous tissue (case 12). **(G)** MAC, ulcers gradually healing, predominantly with fibrous tissue proliferation and minimal inflammatory reaction (case 16). **(H)** *M. haemophilum*, hyperkeratosis of the epidermis, and chronic inflammatory cells were band-like infiltrated in the superficial dermis (case 21). **(I)** *M. avium*, significant inflammatory reaction extending deep into the subcutaneous fat tissue (case 25). **(J)** *M. colombiense*, significant inflammatory reaction with interstitial edema (case 26). **(K)** *M. mantenii*, significant inflammatory reaction around the ulcer, epidermal hyperplasia, and elongation of epithelial ridges (case 27). **(L)** MTB, epidermal hyperplasia, and inflammatory reaction was more pronounced in the superficial dermis.

Supplementary Figure 2 Statistical analysis of the correlation between the positive rates of CD68, CD86, CD163, and CD206 in macrophages in TB. **(A–F)** No statistical significance correlations between CD68 and CD206, CD86 and CD163, CD68 and CD86, CD68 and CD163, CD86 and CD206, and CD163 and CD206.

Supplementary Table 1 Comparison of granuloma types between NTMD and TB. NNG, Non-necrotizing granuloma, CNG, Caseous necrotizing granuloma, NCNG, Non-caseous necrotizing granuloma.

Supplementary Table 2 NTMD pathomorphological characterization of diverse bacterial strains. * Biopsy specimens from the nasopharynx were included, where lymphocytic and plasma cell proliferation may not be directly related to NTM. PH, pseudoepitheliomatous hyperplasia, CAN, caseous necrosis, CON, coagulative necrosis, M, microabscess, NG, necrotizing granuloma, NNG, non-necrotizing granuloma.

Supplementary Table 3 PD-L1 expression of immune cells in NTMD and TB.
