# Supplementary material for: Pathological and immune features of non-tuberculous mycobacteria and Mycobacterium tuberculosis cutaneous/mucosa infections of fifty-four biopsies
Source: Front Cell Infect Microbiol. 2025 Oct 20;15:1664902. doi: 10.3389/fcimb.2025.1664902 (PMC12580264; doi:10.3389/fcimb.2025.1664902)
Supplement: Supplementary Figure 1 — Gross pathological morphological features of NTMD and TB (H&E staining). (A–C) M. marinum. (A) Pseudoepitheliomatous hyperplasia with significant inflammatory reaction (case 7). (B) Significant inflammatory reaction concentrated in the epidermis and superficial dermis (case 8). (C) Pseudoepitheliomatous hyperplasia with marked hyperkeratosis (case 1). D-F M. abscessus. (D) Inflammation was limited to the epidermis and superficial dermis, with relatively mild inflammation (case 13). (E) Significant inflammatory reaction with central necrosis and neutrophil aggregation (case 14). (F) Inflammation was multifocal and extended deep into the subcutaneous tissue (case 12). (G) MAC, ulcers gradually healing, predominantly with fibrous tissue proliferation and minimal inflammatory reaction (case 16). (H) M. haemophilum, hyperkeratosis of the epidermis, and chronic inflammatory cells were band-like infiltrated in the superficial dermis (case 21). (I) M. avium, significant inflammatory reaction extending deep into the subcutaneous fat tissue (case 25). (J) M. colombiense, significant inflammatory reaction with interstitial edema (case 26). (K) M. mantenii, significant inflammatory reaction around the ulcer, epidermal hyperplasia, and elongation of epithelial ridges (case 27). (L) MTB, epidermal hyperplasia, and inflammatory reaction was more pronounced in the superficial dermis. [file DataSheet1.docx]

Supplemental Digital Content: 2 figures, 3 tables.

**
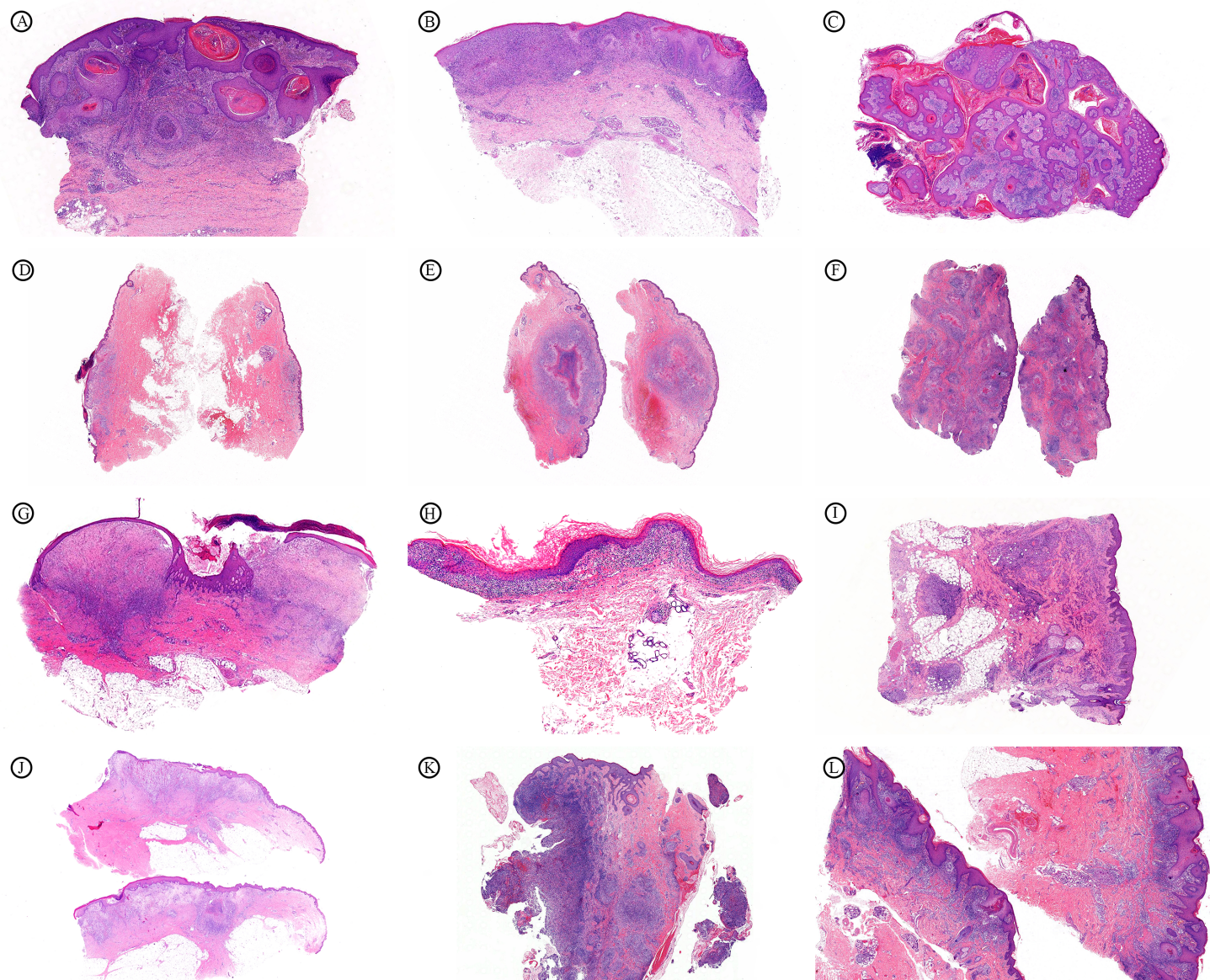
**

**Figure S1** Gross pathological morphological features of NTMD and TB (H&E staining). A-C *M. marinum*. A Pseudoepitheliomatous hyperplasia with significant inflammatory reaction (case 7). B Significant inflammatory reaction concentrated in the epidermis and superficial dermis (case 8). C Pseudoepitheliomatous hyperplasia with marked hyperkeratosis (case 1). D-F *M. abscessus*. D Inflammation was limited to the epidermis and superficial dermis, with relatively mild inflammation (case 13). E Significant inflammatory reaction with central necrosis and neutrophil aggregation (case 14). F Inflammation was multifocal and extended deep into the subcutaneous tissue (case 12). G MAC, ulcers gradually healing, predominantly with fibrous tissue proliferation and minimal inflammatory reaction (case 16). H *M. haemophilum*, hyperkeratosis of the epidermis, and chronic inflammatory cells were band-like infiltrated in the superficial dermis (case 21). I *M. avium*, significant inflammatory reaction extending deep into the subcutaneous fat tissue (case 25). J *M. colombiense*, significant inflammatory reaction with interstitial edema (case 26). K *M. mantenii*, significant inflammatory reaction around the ulcer, epidermal hyperplasia, and elongation of epithelial ridges (case 27). L MTB, epidermal hyperplasia, and inflammatory reaction was more pronounced in the superficial dermis.


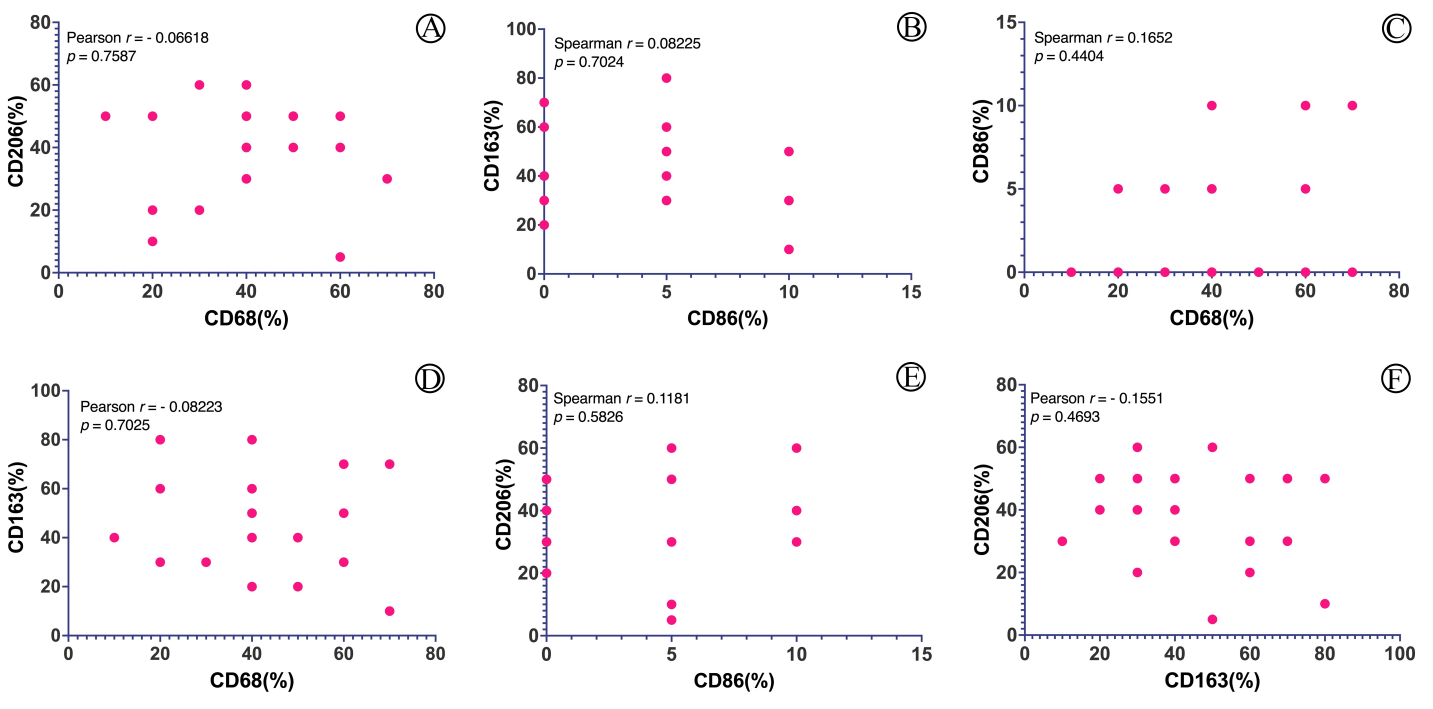


**Figure S2** Statistical analysis of the correlation between the positive rates of CD68, CD86, CD163, and CD206 in macrophages in TB. A-F No statistical significance correlations between CD68 and CD206, CD86 and CD163, CD68 and CD86, CD68 and CD163, CD86 and CD206, and CD163 and CD206.

**Table S1. Comparison of granuloma types between NTMD and TB**

| Granuloma | NTMD（n=27） Number of positive case (percentage positive) | | | |  | TB（n=27） Number of positive case (percentage positive) | | | | Adj.*P* |
| --- | --- | --- | --- | --- | --- | --- | --- | --- | --- | --- |
|  | None | NNG | CNG | NCNG |  | None | NNG | CNG | NCNG |  |
| Granuloma | 8（29.6%） | 11（40.7%） | 4（14.8%) | 4（14.8%) |  | 1（3.7%) | 4（14.8%) | 12（44.4%) | 10（37.0%) | < 0.001 |
| NNG, Non-necrotizing granuloma, CNG, Caseous necrotizing granuloma, NCNG, Non-caseous necrotizing granuloma. | | | | | | | | | | |

**Table S2. NTMD pathomorphological characterization of diverse bacterial strains**

| Pathological Feature | *M.marinum*（n=8） | *M.abscessus*（n=6） | MAC（n=5） | *M.haemophilum*（n=3） | *M. avium*（n=3） | *M.colombiense*（n=1） | *M.mantenii*（n=1） |
| --- | --- | --- | --- | --- | --- | --- | --- |
| Epidermal hyperplasia | None/mild; PH | None/mild | None/mild | None/mild | None/mild | Moderate | Mild |
| Ulceration | None | None/present | None/present | None | None | Present | Present |
| Hyperkeratosis | Mild to marked | None/mild | Mild to marked | Mild | None/mild | Mild | None |
| Parakeratosis | None | None | Mild to marked | None | None | Mild | None |
| Granulocytic exudates | None/mild | None/significant | None/significant | None | None/mild | Mild | Mild |
| Plasma exudates | None | None | None/mild | None/mild | None/mild | None/mild | None/mild |
| Spongiosis | None/mild | None/mild | None | None | None/mild | None | None |
| Appendage destruction | Present | None/present | None/present | None/present | Present | Present | Present |
| Hemorrhage | None/focal | None/focal | None/focal | None/focal | None/focal | None | Focal |
| Microvessels hyperplasia | Mild to marked | None/mild | None/mild | None/mild | None/mild | Moderate | Mild |
| Microvessels dilation and congestion | Present | None/present | Present | None | None/present | None | Mild |
| Vasculitis | None | None | None | None | None | None | None |
| Necrosis | CAN/CON | None/CAN | None/CON | None/CAN/CON | None/CON | CON | None |
| Interstitial edema | Mild to marked | None/mild | Mild to marked | Mild | None/mild | Marked | None |
| Lymphocytic proliferation | Multifocal/expansive | Focal/expansive | Focal/multifocal | Focal/multifocal | Focal/multifocal* | Expansive | Expansive |
| Plasma cells | Focal/multifocal | Focal/expansive | Focal/expansive | Focal | Focal/multifocal* | Focal | Expansive |
| Eosinophils | None/focal | None/multifocal | None/focal | None/focal | None/multifocal | None | None |
| Neutrophils | Focal/multifocal，M | Focal/multifocal，M | Focal/multifocal，M | Focal/multifocal | Focal | Multifocal，M | Multifocal |
| Multinucleated giant cells | Focal/multifocal | None/multifocal | None/focal | None/focal | None/focal | Multifocal | None |
| Histiocytes | Focal/multifocal | Focal/expansive | Focal/expansive | Focal | Multifocal | Focal | Focal |
| Granuloma | NG/NNG | None/NG/NNG | None/NG/NNG | None/NG | NG/NNG | NG | None |
| * Biopsy specimens from the nasopharynx were included, where lymphocytic and plasma cell proliferation may not be directly related to NTM.  PH, pseudoepitheliomatous hyperplasia, CAN, caseous necrosis, CON, coagulative necrosis, M, microabscess, NG, necrotizing granuloma, NNG, non-necrotizing granuloma. | | | | | | | |

**Table S3. PD-L1 expression of immune cells in NTMD and TB**

| PD-L1 Positivity rate.（%） | NTMD（n=24) | TB (n=24) | Adj.*P* |
| --- | --- | --- | --- |
| PD-L1 Low Expression（<10%) | 18(75%) | 13(54.2%) | 0.227 |
| PD-L1High Expression（≥10%) | 6(25%) | 11(45.8%) |  |
